# Supplementary material for: Pharmaceutical Payments to Japanese Board-Certified Infectious Disease Specialists: A Four-Year Retrospective Analysis of Payments from 92 Pharmaceutical Companies between 2016 and 2019
Source: Int J Environ Res Public Health. 2022 Jun 16;19(12):7417. doi: 10.3390/ijerph19127417 (PMC9223711; doi:10.3390/ijerph19127417)
Supplement: Supplementary file 1 [file ijerph-19-07417-s001.zip › ijerph-1724180-supplementary.pdf]

Figure S1. Distribution of payment values per specialist

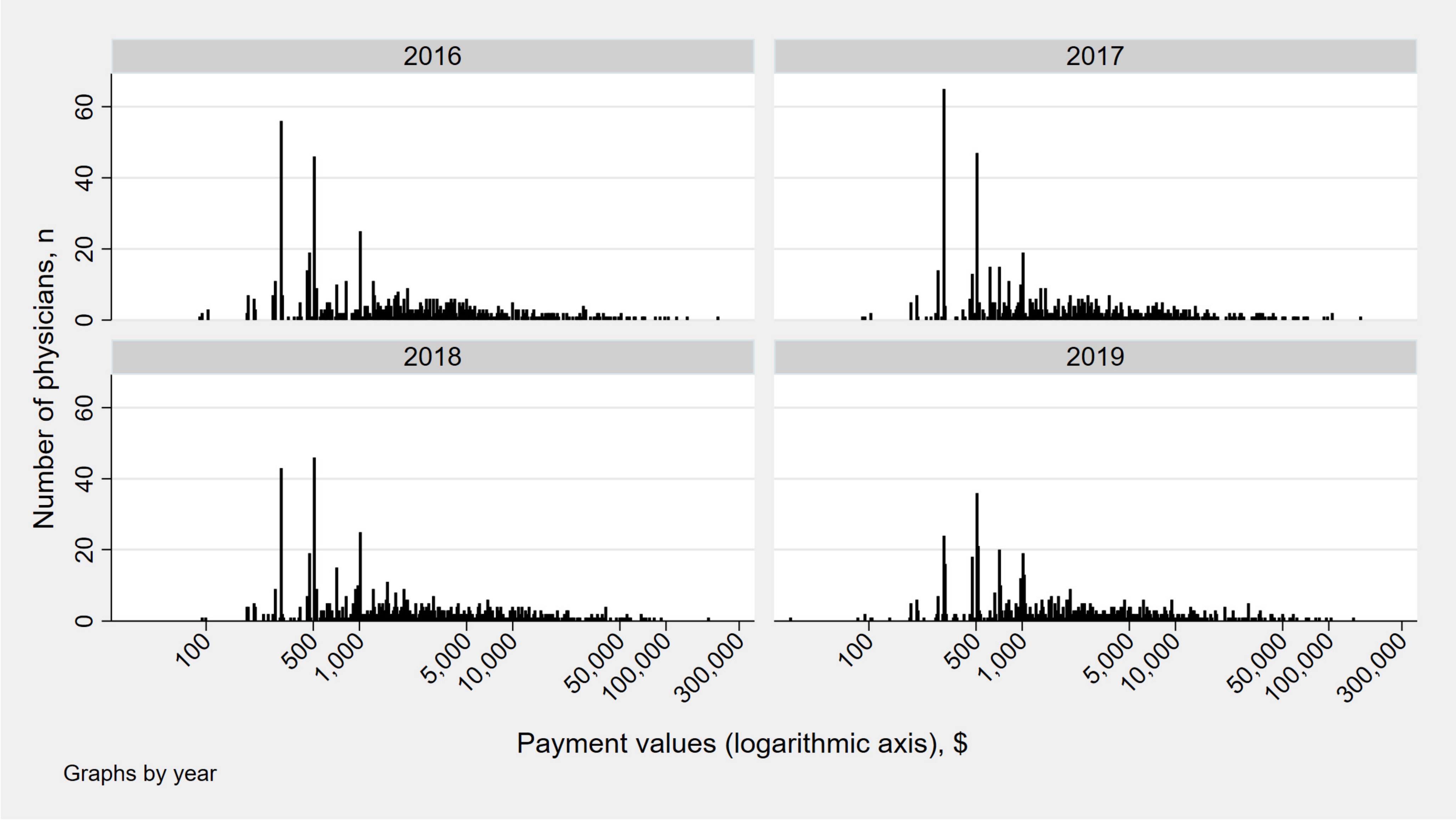

Figure S2. Payment concentration

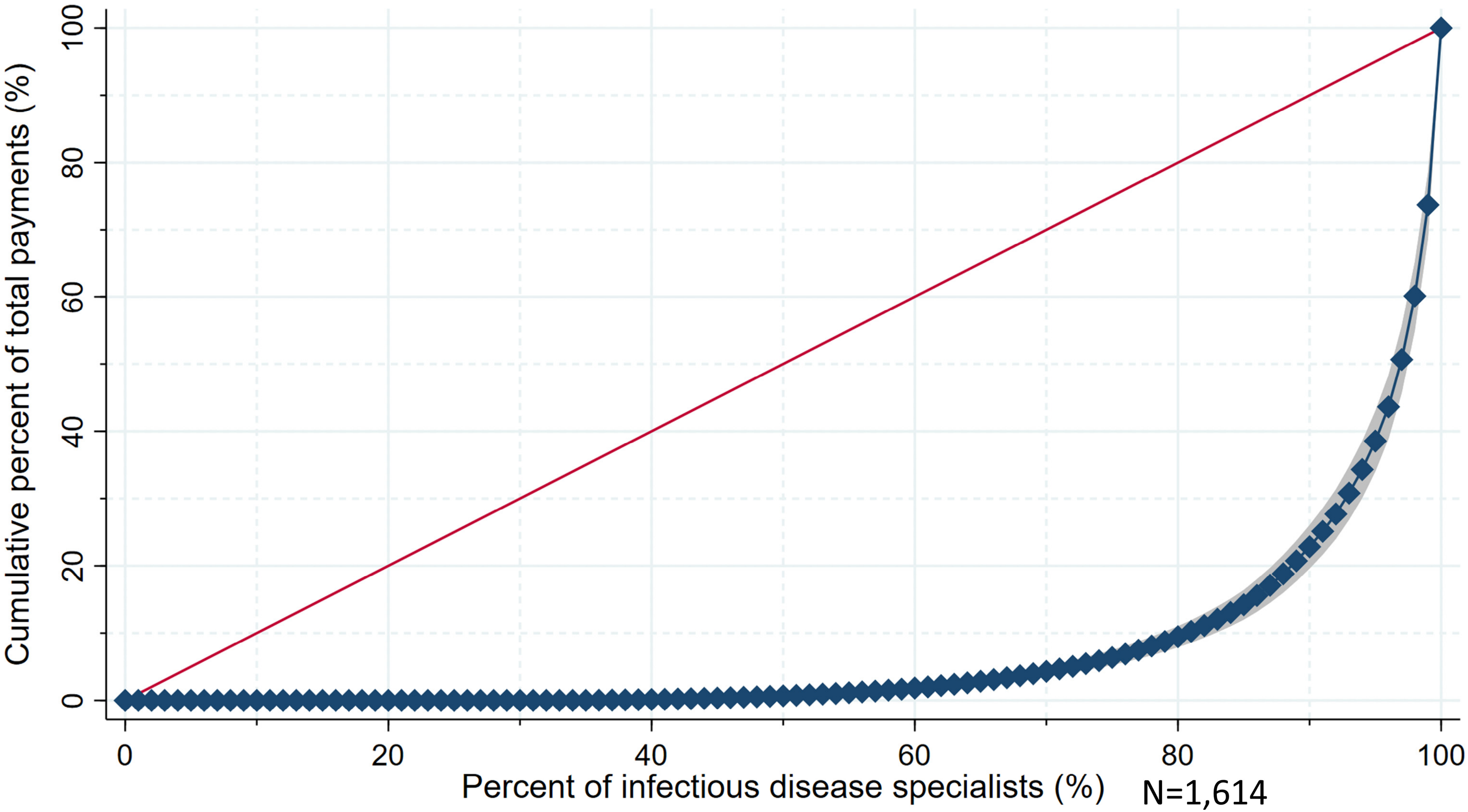

Figure S3. Payment category by company

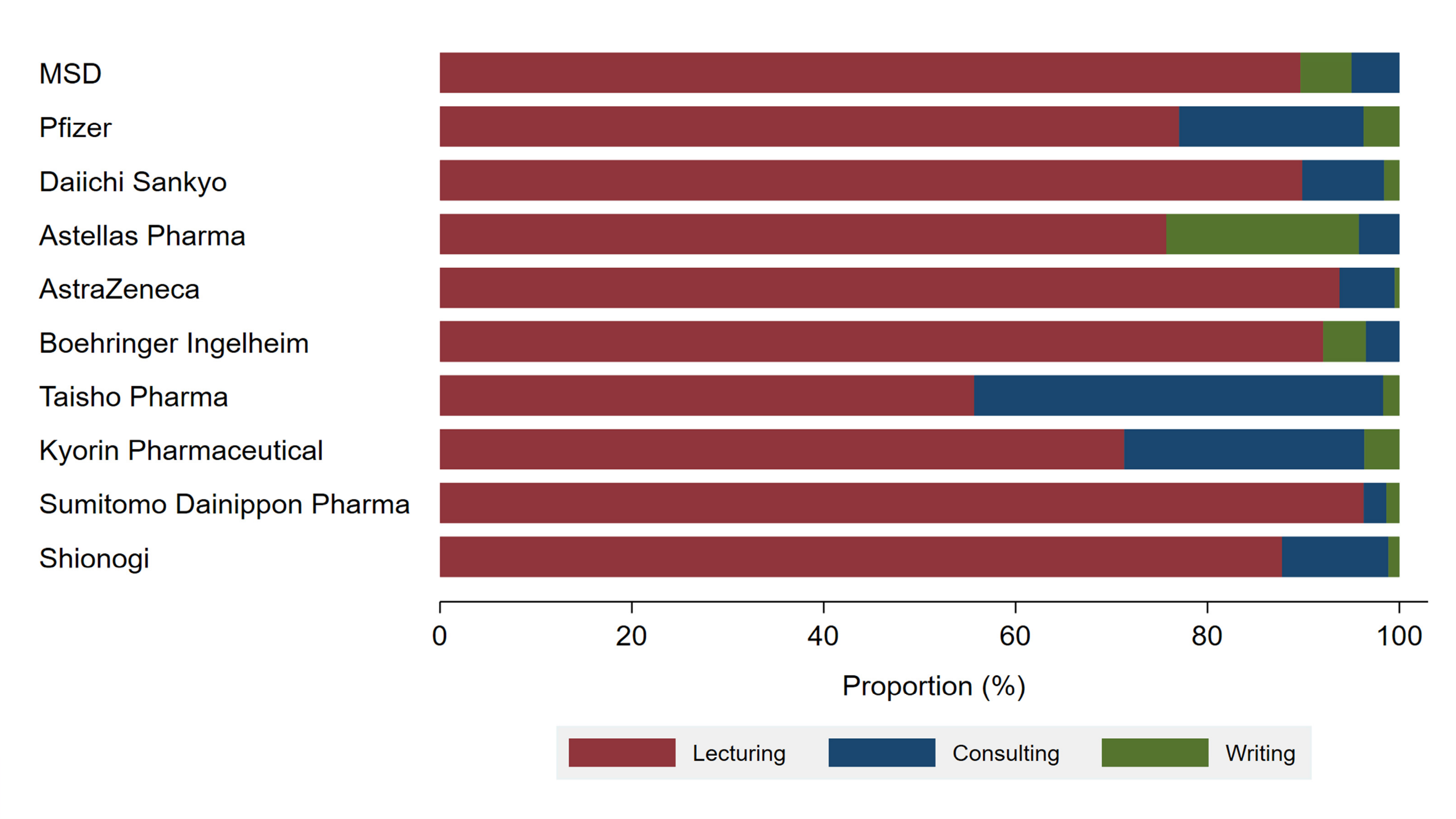

Table S1. New and additional indications for infectious diseases in Japan between 2015 and 2019

Brand name	Name	Pharmaceutical companies	Approval date	Price per drug unit, $*	Indication	Category
**ZERBAXA**	Ceftolozane sulfate/Tazobactam sodium	Manufacturer and distributor: MSD K. K	December 20, 2019	$59 (1.5g/bottle)	Treatment of sepsis and pneumonia caused by Serratia and Hemophilus influenza	Additional indication
**LASVIC**	Lascufloxacin hydrochloride	Manufacturer and distributor: Kyorin Pharmaceuticals	September 20, 2019	$3 (75mg/pill)<br>$37 (150mg/ intravenous infusion kit)	Treatment of laryngopharyngitis, tonsillitis, acute bronchitis, pneumonia, secondary infection of chronic respiratory disease, otitis media and sinusitis	New approval
**MAVIRET**	Glecaprevir hydrate/Pibrentasvir	Manufacturer and distributor: AbbVie GK	August 22, 2019	$168/pill	Improvement of viremia in patients with chronic hepatitis C or compensated cirrhosis type C	Additional indication
**AZIMYCIN**	Azithromycin hydrate	Manufacturer and distributor: Senju Pharmaceuticals<br>Distributor: Takeda Pharmaceutical	June 18, 2019	$3 (1%1ml eye-drops)	Treatment of conjunctivitis, blepharitis, hordeolum and dacryocystitis	New approval
**SYMTUZA**	Darunavir ethanolate/Cobicistat /Emtricitabine/Tenofovir alafenamide fumarate	Manufacturer and distributor: Janssen Pharmaceutical K. K	June 18, 2019	$44/pill	Treatment of HIV-1 infection	New approval
**INAVIR**	Laninamivir octanoate hydrate	Manufacturer and distributor: Daiichi Sankyo	June 18, 2019	$20 (20mg/inhalation kit)<br>$39 (160mg/bottle)	Treatment of influenza A or B virus infection	New approval
**GENVOYA**	Elvitegravir/Cobicistat /Emtricitabine/Tenofovir alafenamide fumarate	Manufacturer and distributor: Gilead Sciences	May 22, 2019	$65/pill	Treatment of HIV-1 infection	Additional indication
**BIKTARVY**	Bictegravir sodium/ Emtricitabine/Tenofovir alafenamide fumarate	Manufacturer and distributor: Gilead Sciences	March 26, 2019	$65/pill	Treatment of HIV-1 infection	New approval
**ZERBAXA**	Ceftolozane sulfate/Tazobactam sodium	Manufacturer and distributor: MSD K. K	January 8, 2019	$59 (1.5g/bottle)	Treatment of cystitis, pyelonephritis, peritonitis, intra-abdominal abscess, cholecystitis and liver abscess	New approval
**EPCLUSA**	Sofosbuvir/Velpatasvir	Manufacturer and distributor: Gilead Sciences	January 8, 2019	$562/pill	Improvement of viremia in patients with chronic hepatitis C or compensated cirrhosis type C who have previously been treated.<br>Improvement of viremia in patients with decompensated cirrhosis type C	New approval
**REBETROL**	Ribavirin	Manufacturer and distributor: ViiV Healthcare<br>Distributor: GlaxoSmithKline K. K<br>Promotional partner: Shionogi	January 8, 2019	$4 (200mg)	Improvement of viremia in patients with chronic hepatitis C or compensated cirrhosis type C who have previously been treated	Additional indication
**JULUCA**	Dolutegravir sodium/Rilpivirine hydrochloride	Manufacturer and distributor: ViiV Healthcare	November 26, 2018	$50/pill	Treatment of HIV-1 infection	New approval

		Distributor: GlaxoSmithKline K. K				
**ODEFSEY**	Rilpivirine hydrochloride /Emtricitabine/Tenofovir alafenamide fumarate	Manufacturer and distributor: Janssen Pharmaceutical K. K	August 21, 2018	$56/pill	Treatment of HIV-1 infection	New approval
**ISENTRESS**	Raltegravir potassium	Manufacturer and distributor: MSD K. K	May 14, 2018	$15 (400mg/pill)<br>$15 (600mg/pill)	Treatment of HIV infection	New approval
**KAKETSUKEN**	Emulsion-adjuvanted cell-culture derived influenza HA vaccine (H5N1)	Manufacturer and distributor: The Chemo-Sero-Therapeutic Research Institute	March 23, 2018	NA	Prevention of pandemic influenza (H5N1)	Additional indication
**KAKETSUKEN**	Emulsion-adjuvanted cell-culture derived influenza HA vaccine (prototype)	Manufacturer and distributor: The Chemo-Sero-Therapeutic Research Institute	March 23, 2018	NA	Prevention of pandemic influenza	Additional indication
**XOFLUZA**	Baloxavir marboxil	Manufacturer and distributor: Shionogi	February 23, 2018	$14 (10mg/pill)<br>$22 (20mg/pill)	Treatment of influenza A or B virus infections	New approval
**HARVONI**	Ledipasvir acetonate/Sofosbuvir	Manufacturer and distributor: Gilead Sciences	February 16, 2018	$509/pill	Improvement of viremia in patients with chronic hepatitis C or compensated cirrhosis type C in serogroup 2	Additional indication
**ZINPLAVA**	Bezlotoxumab	Manufacturer and distributor: MSD K. K	September 27, 2017	$3081 (625mg/25ml bottle)	Prevention of recurrent Clostridium difficile infection	New approval
**MAVIRET**	Glecaprevir hydrate/ Pibrentasvir	Manufacturer and distributor: AbbVie GK	September 27, 2017	$168/pill	Improvement of viremia in patients with chronic hepatitis C or compensated cirrhosis type C	New approval
**TAMIFLU**	Oseltamivir phosphate	Manufacturer and distributor: Chugai Pharmaceutical	March 24, 2017	$2 (75mg/pill)<br>$2 (3%/g dry syrup)	Treatment of influenza A or B virus infection	Additional indication
**SOVALDI**	Sofosbuvir	Manufacturer and distributor: Gilead Sciences	March 24, 2017	$395 (400mg/pill)	Improvement of viremia in patients with chronic hepatitis C or compensated cirrhosis type C in neither Serogroup 1 (genotype 1) nor Serogroup 2 (genotype 2)	Additional indication
**REBETOL**	Ribavirin	Manufacturer and distributor: MSD K. K	March 24, 2017	$4 (200mg/pill)	Improvement of viremia in patients with chronic hepatitis C or compensated cirrhosis type C in neither Serogroup 1 (genotype 1) nor Serogroup 2 (genotype 2)	Additional indication
**OZEX**	Tosufloxacin tosilate hydrate	Manufacturer and distributor: Fujifilm Toyama Chemical	March 2, 2017	$1.1 (60mg/pill)<br>$0.5 (75mg/pill)<br>$0.6 (150mg/pill)<br>$1.2 (0.3%/ml eye-drops)	Treatment of mycoplasma pneumonia caused by Mycoplasma pneumoniae	Additional indication
**RIAMET**	Artemether/Lumefantrine	Manufacturer and distributor: Novartis Pharma K. K	December 19, 2016	$2/pill	Treatment of malaria	New approval
**VAXEM HIB**	Hemophilus influenzae type b vaccine adsorbed	Manufacturer and distributor: Takeda Pharmaceutical	December 19, 2016	NA	Prophylaxis of Hemophilus influenzae type b infections	Additional indication
**XIMENCY**	Daclatasvir hydrochloride/ Asunaprevir/Beclabuvir	Manufacturer and distributor: Bristol Myers Squibb	December 19, 2016	$99/pill	Improvement of viremia in patients with chronic hepatitis C or compensated cirrhosis type C in	New approval

	hydrochloride				serogroup 1 (genotype 1)	
**DESCOVY**	Emtricitabine/ Tenofovir alafenamide fumarate	Manufacturer and distributor: Gilead Sciences	December 9, 2016	$26/pill (LT)<br>$37/pill (HT)	Treatment of HIV-1 infection	New approval
**PREZCOBIX**	Darunavir ethanolate/ Cobicistat	Manufacturer and distributor: Janssen Pharmaceutical K. K	November 22, 2016	$19	Treatment of HIV infection	New approval
**ERELSA**	Elbasvir	Manufacturer and distributor: MSD K. K	September 28, 2016	$223 (50mg/pill)	Improvement of viremia in patients with chronic hepatitis C or compensated cirrhosis type C in serogroup 1 (genotype 1)	New approval
**VIEKIRAX**	Ombitasvir hydrate/ Paritaprevir hydrate/ Ritonavir	Manufacturer and distributor: AbbVie GK	September 28, 2016	$204/pill	Improvement of viremia in patients with chronic hepatitis C in serogroup 2 (genotype 2)	Additional indication
**GRAZYNA**	Grazoprevir hydrate	Manufacturer and distributor: MSD K. K	September 28, 2016	$80 (50mg/pill)	Improvement of viremia in patients with chronic hepatitis C or compensated cirrhosis type C in serogroup 1 (genotype 1)	New approval
**REBETOL**	Ribavirin	Manufacturer and distributor: MSD K. K	September 28, 2016	$4 (200mg/capsule)	Improvement of viremia in patients with chronic hepatitis C in serogroup 2	Additional indication
**INAVIR**	Laninamivir octanoate hydrate	Manufacturer and distributor: Daiichi Sankyo	August 26, 2016	$20 (20mg/inhalation kit)<br>$39 (160mg/bottle)	Prophylaxis of influenza A or B virus infections	Additional indication
**GENVOYA**	Elvitegravir/Cobicistat/ Emtricitabine/Tenofovir alafenamide fumarate	Manufacturer and distributor: Gilead Sciences	June 17, 2016	$65/pill	Treatment of HIV-1 infection	New approval
**MALARONE**	Atovaquone/Proguanil hydrochloride	Manufacturer and distributor: GlaxoSmithKline K. K	March 28, 2016	$5/pill (adult)<br>$2/pill (children)	Treatment and prevention of malaria	Additional indication
**PRIMAQUINE**	Primaquine phosphate	Manufacturer and distributor: Sanofi S.A.	March 28, 2016	$21 (15mg/pill)	Treatment of malaria caused by Plasmodium vivax and P. ovale	New approval
**BIKEN**	Freeze-dried live attenuated varicella vaccine	Manufacturer and distributor: The Research Foundation for Microbial Diseases of Osaka University<br>Distributor: Mitsubishi Tanabe Pharma Corporation	March 18, 2016	NA	Prevention of herpes zoster in individuals 50 years of age and older	Additional indication
**KITASATO DAIICHI SANKYO**	Adsorbed cell culture-derived influenza vaccine (H5N1)	Manufacturer and distributor: Daiichi Sankyo<br>Distributor: Kitasato Pharmaceutical Industry	March 18, 2016	NA	Prevention of pandemic influenza (H5N1)	Additional indication
**TRIBIK**	Adsorbed diphtheria-purified pertussis-tetanus combined vaccine	Manufacturer and distributor: The Research Foundation for Microbial Diseases of Osaka University<br>Distributor: Mitsubishi Tanabe	February 29, 2016	NA	Prevention of pertussis, diphtheria and tetanus	Additional indication

		Pharma Corporation				
**VAXEM HIB**	Hemophilus influenzae type b vaccine absorbed	Manufacturer and distributor: Takeda Pharmaceutical	January 22, 2016	NA	Prophylaxis of Hemophilus influenzae type b infections	New approval
**REMICADE**	Infliximab	Manufacturer and distributor: Mitsubishi Tanabe Pharma Corporation<br>Distributor: Janssen Biotech	December 21, 2015	$648(100mg bottle)	Treatment of acute-phase Kawasaki's disease in patients who have not responded sufficiently to conventional therapies	Additional indication
**VIEKIRAX**	Ombitasvir hydrate/Paritaprevir hydrate/Ritonavir	Manufacturer and distributor: AbbVie GK	September 28, 2015	$204/pill	Improvement of viremia in patients with chronic hepatitis C or compensated cirrhosis type C in serogroup 1 (genotype 1)	New approval
**CIPROXAN**	Ciprofloxacin	Manufacturer and distributor: Bayer Yakuhin	September 24, 2015	$0.3 (100mg/pill)<br>$0.4 (200mg/pill)<br>$17 (200mg/100ml)<br>$21 (300mg/150ml)<br>$19 (400mg/200ml)	Treatment of sepsis, pneumonia, etc.	Additional indication
**HARVONI**	Ledipasvir acetonate/Sofosbuvir	Manufacturer and distributor: Gilead Sciences	July 3, 2015	$509/pill	Improvement of viremia in patients with chronic hepatitis C or compensated cirrhosis type C in serogroup 1 (genotype 1)	New approval
**DIFLUCAN**	Fluconazole	Manufacturer and distributor: Pfizer	May 26, 2015	$3 (50mg capsule)<br>$4 (100mg capsule)<br>$17 (0.1% 50ml bottle)<br>$22 (0.2% 50ml bottle)<br>$36 (0.2% 100ml bottle)	Treatment of vaginitis and vulvovaginitis caused by Candida	Additional indication
**ALDREB**	Colistin sodium methanesulfonate	Manufacturer and distributor: GlaxoSmithKline K. K	March 26, 2015	$77 (150mg bottle)	Treatment of infections caused by colistin-sensitive Escherichia coli, Citrobacter, Klebsiella, Enterobacter, Pseudomonas aeruginosa and Acinetobacter	New approval
**SYNFLORIX**	Pneumococcal 10-valent conjugate vaccine adsorbed	Manufacturer: GlaxoSmithKline K. K<br>Distributor: Daiichi Sankyo	March 26, 2015	NA	Prophylaxis of pneumonia and pneumococcal invasive diseases	New approval
**KAKETSUKEN**	Cell culture-derived influenza emulsion HA vaccines (prototype)	Manufacturer and distributor: The Chemo-Sero-Therapeutic Research Institute	March 26, 2015	NA	Prevention of pandemic influenza	New approval
**SOVALDI**	Sofosbuvir	Manufacturer and distributor: Gilead Sciences	March 26, 2015	$395 (400mg pill)	Improvement of viremia in patients with chronic hepatitis C or compensated cirrhosis type C in serogroup 2 (genotype 2)	New approval
**COPEGUS**	Ribavirin	Manufacturer and distributor: Chugai Pharmaceutical	March 26, 2015	$6 (200mg/pill)	Improvement of viremia with the concomitant use of sofosbuvir in patients with chronic hepatitis C or compensated cirrhosis type C in serogroup 2 (genotype 2)	Additional indication
**DAKLINZA**	Daclatasvir hydrochloride	Manufacturer and distributor: Bristol Myers Squibb	March 20, 2015	$74 (60mg/pill)	Improvement of viremia in patients with chronic hepatitis C or compensated cirrhosis type C in serogroup 1 (genotype 1)	Changed approval

**TRIUMEQ**	Dolutegravir sodium, Abacavir sulfate, Lamivudine	Manufacturer and distributor: ViiV Healthcare	March 16, 2015	$64/pill	Treatment of HIV infection	New approval
**VENOGLOBULIN**	Polyethylene glycol treated human normal immunoglobulin	Manufacturer and distributor: Japan Blood Products Organization	February 2, 2015	$347 (5g/100ml bottle)<br>$702 (10g/200ml bottle)	Prevention of acute otitis media, acute bronchitis, or pneumonia caused by Pneumococcus or Hemophilus influenzae in patients associated with a decrease in serum IgG2 levels	Additional indication

*Price per drug was converted into US dollars using the 2019 average monthly exchange rates of ¥109.0 per $1.

Price per drug was used as of February 19, 2022.

Drug price for vaccines was not determined and was open priced in Japan. So, it was not available.

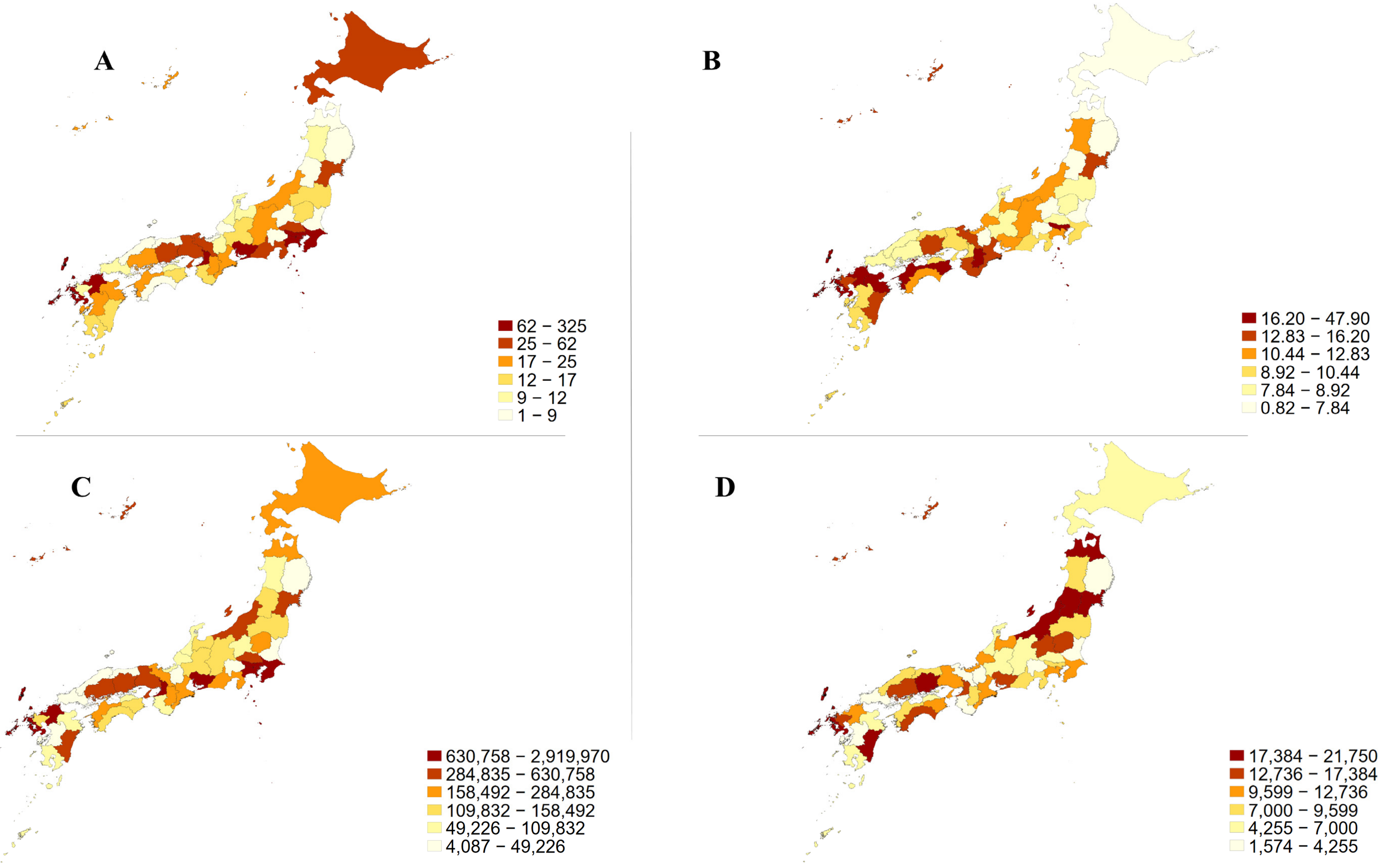

Figure S4. Geographical characteristics of payment distribution

5A: The number of infectious disease specialists in 2021; 5B: The number of infectious disease specialists per million population in 2021; 5C: total personal payment values to the infectious disease specialists from 2016 to 2019; 5D: average personal payment values per infectious disease specialist from 2016 to 2019
